# The rate of in vitro fludarabine-induced peripheral blood and bone marrow cell apoptosis may predict the chemotherapy outcome in patients with chronic lymphocytic leukemia

**DOI:** 10.1007/s00228-015-1893-0

**Published:** 2015-07-05

**Authors:** Monika Podhorecka, Piotr Klimek, Sylwia Chocholska, Agnieszka Szymczyk, Arkadiusz Macheta, Malgorzata Kowal, Anna Dmoszynska, Marek Hus

**Affiliations:** Department of Haematooncology and Bone Marrow Transplantation, Medical University of Lublin, Staszica 11, 20-081 Lublin, Poland

**Keywords:** CLL, Apoptosis, Cell culture, Fludarabine

## Abstract

**Purpose:**

The problem of drug sensitivity and predicting the outcome of chemotherapy seems to be of great importance in hemato-oncological disorders. There are some factors that can help to predict effects of chemotherapy in chronic lymphocytic leukemia (CLL), such as presence of del17p, del11q, or *TP53* gene mutations, which result in resistance to purine analogues and alkylating drugs. Despite the new therapeutic options introduced recently, purine analogues in combination with cyclophosphamide and the monoclonal antibody rituximab is still the gold standard for the first-line treatment of fit patients with CLL. The aim of this study was to assess whether the rate of apoptosis caused by one of purine analogues—fludarabine in cell cultures differs between patients who clinically respond to fludarabine-based chemotherapy and those who do not respond.

**Methods:**

CLL leukemic cells, obtained from peripheral blood and bone marrow of 23 patients, were cultured in the presence of fludarabine. After 24 h of incubation, the rate of apoptosis, indicated by the expression of active caspase-3, was assessed with flow cytometry and then analyzed regarding clinical response to fludarabine-based regimens.

**Results:**

The percentage of apoptotic cells induced by fludarabine was significantly higher in the group of patients who achieved remission in comparison to the group with no response to purine analogues therapy. Interestingly, we observed that among the patients who did not respond to chemotherapy, the presence of del17p and del11q was detected only once. Other non-responders had no detectable genetic abnormalities.

**Conclusions:**

Based on these results, it can be presumed that in vitro drug sensitivity test, which is easy to perform, may predict the outcome of fludarabine-based chemotherapy in CLL patients.

**Electronic supplementary material:**

The online version of this article (doi:10.1007/s00228-015-1893-0) contains supplementary material, which is available to authorized users.

## Introduction

Chronic lymphocytic leukemia (CLL) is characterized by the accumulation of malignant CD19+/CD5+ B cells in blood, bone marrow, and lymphoid organs [1–3]. The leukemic transformation is initiated by genomic alterations causing the deletion of specific micro-RNA genes and increasing the resistance of B cells towards apoptosis [1, 4]. The biology of CLL is also directly entwined with its microenvironment, in which accessory cells can promote leukemia cell growth and survival. Recently, much attention has been focused on the B cell receptor (BCR) and on chemokine receptors that enable CLL cells to home to lymphoid tissues and to establish the leukemia microenvironment [5]. BCR signaling plays an important pathogenic role, based on structural restrictions of the BCR, and BCR-dependent survival and growth of the malignant B cells. In CLL, ligand-independent and ligand-dependent BCR signaling has been characterized. They can involve mutations of BCR pathway components or be triggered by antigens present in the tissue microenvironment [6].

CLL typically occurs in elderly patients and has a highly variable clinical course [7]. Some patients have indolent disease and never need treatment, but in others, the clinical course is aggressive and soon after diagnosis requires intensive treatment [8, 9]. This variable clinical course of CLL makes the role of prognostic factors very important, especially for distinguishing the group of patients who require intensive treatment from those who will benefit from milder forms of therapy. A number of markers of prognostic relevance have been identified for CLL. Among these markers, biological risk factors such as the VH mutation status or its surrogate markers as well as genomic aberrations may have the power to identify the subgroup of patients with poor prognosis from early stage patients [9, 10]. Among them deletions of the short arm of chromosome 17 (del(17p)) or *TP53* gene mutation predict resistance to most available therapies [7].

Recently, substantial strides have been made in the treatment of CLL. The novel class of targeted therapies, such as two oral tyrosine kinase inhibitors, ibrutinib (Bruton’s kinase inhibitor) and idelalisib (phosphatidylinositol 3-kinase delta inhibitor), have received approval for the treatment of CLL patients. Another promising drugs are BCL-2 protein antagonists that induce apoptosis in leukemic cells. The new monoclonal antibody—obinutuzumab, has also been recently introduced to the therapy of older patients in combination with chlorambucil. Despite the new therapeutic options, purine analogues in combination with cyclophosphamide and the monoclonal antibody rituximab is still the gold standard for the first-line treatment of fit patients with CLL [11]. Some patients, however, are refractory to purine analogues, especially those with del17p or del11q. The presence of other, not explored yet, factors may be responsible for therapy resistance as well. Thus, exploring the process of drug-induced apoptosis in vitro may be of great importance for predicting the susceptibility of leukemic cells to the drug, especially if it would reflect this susceptibility in vivo.

In this study, we attempted to assess the rate of CLL cell apoptosis caused by one of purine analogues—fludarabine. The experiments were performed in short-term cell cultures of peripheral blood and bone marrow supplemented with this drug. We analyzed the percentage of cells with active caspase-3 expression. Then, the apoptosis rate was assessed as a marker predicting the outcome of chemotherapy.

## Materials and methods

### Patients

The study was approved by the local ethical committee. Peripheral blood and bone marrow samples were obtained from 23 patients after informed consent. Diagnosis of CLL was based on a clinical examination and morphological and immunological criteria. The patients were enrolled to the study prior to the onset of therapy with fludarabine-based regimens and were not treated previously with any chemotherapeutic agents.

### Cell culture

Mononuclear cells were isolated by density gradient centrifugation using Lymphoprep (Nycomed, Norway). Then, the cells were cultured in medium consisting of RPMI 1640 with 2 mM l-glutamine, 100 units/ml penicillin, 100 μg/ml streptomycin, and 10 % fetal calf serum (FCS) at a final concentration of 2 × 10^6^ cells/ml. This culture medium was supplemented with fludarabine (Schering AG, Germany) at a concentration of 1 μg/ml. We selected such a concentration of fludarabine on the basis of our preliminary experiments in CLL cultures, in which this concentration induced significant number of apoptosis with spontaneous apoptosis that did not exceed 50 % of the total cell culture [12]. The cells were cultured at 37 °C in a 5 % CO_2_ atmosphere, and they were exposed to the drugs for 24 h. Respective cell samples incubated in the absence of any drug for periods of time equivalent to the drug-treated cells were used as a negative control.

### Immunocytochemical detection of active caspase-3 as a marker of apoptosis

The blood and bone marrow samples that had been treated in culture with fludarabine (∼10^5^ cells) were initially incubated for 15 min with anti-CD19 PerCP and anti-CD5 APC-conjugated monoclonal antibody (MoAb) (DAKO, Denmark) at room temperature. After fixation and permeabilization procedures (IntraPrep kit, Immunotech, France), the cells were incubated with anti-active caspase-3 MoAb, PE conjugated (Pharmingen, USA), or an isotype-matched negative control (Dako, Denmark) in darkness at room temperature for 15 min. After washing, the labelled cells were analyzed by multi-parameter flow cytometry.

### Fluorescence measurement

**Fig. 1:**
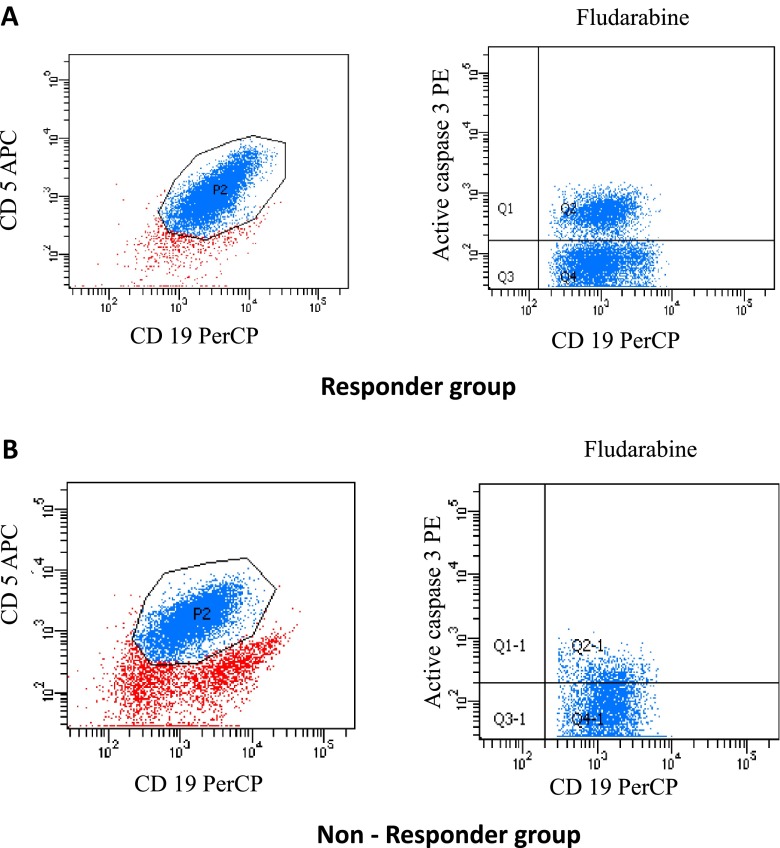
The representative flow cytometry dot plots showing expression of caspase-3 by CD19+/CD5+ cells of peripheral blood in CLL patients that were induced to undergo apoptosis by fludarabine. **a** Patient of responder group with response to fludarabine-based chemiotherapy in vivo. **b** Patient of non-responder group with no response to fludarabine-based chemotherapy in vivo

The samples were measured with a FACSCalibur (Becton Dickinson) using standard emission filters for green and red fluorescence and CellQuest software. The CD5+/CD19+ population was initially gated and further analysis was carried out for this population. Ten thousand cells were measured per sample. To determine the frequency of apoptosis, the percentage of active caspase-3 cells, corresponding to that in the control cells, was calculated. Intra-assay variation of the flow cytometry test was assessed using two acquisitions per assay, while inter-assay variation was assessed by performing in the chosen cases two cell cultures of the same patient on different days. Both intra- and inter-assay variation were lower than 10 %. Representative dot plots illustrating the expression of caspase-3 by CD19+/CD5+ CLL cells in cultures with fludarabine are presented in Fig. 1.

### Flow cytometric detection of ZAP-70 and CD38 as prognostic factors

A total of 1 × 10^6^ peripheral blood cells were stained with the monoclonal antibodies CD19 PE (BD Pharmingen), CD5 CyChrome (Caltag Laboratories, USA), or CD3 PE (BD Pharmingen). Following membrane staining, the cells were fixed in 1 % paraformaldehyde solution in PBS for 15 min at room temperature and permeabilized with 70 % ethanol for one hour at −20 °C. After washing with PBS, anti-ZAP-70 antibody (Biomol Research Laboratories, USA) labelled by the ZenonTM Alexa Fluor® 488 Mouse IgG2a Labeling Kit (Molecular Probes, USA) was added to the sample tubes. The samples were incubated with the reagent for 30 min at room temperature, washed once with PBS, and analyzed by flow cytometry (FACSCalibur, Becton Dickinson).

To assess CD38 expression, peripheral blood mononuclear cells were stained with CD38 FITC, CD19 PE, CD5 CyChrome, or IgG1 isotypic control MoAbs. The cells were incubated for 20 min at room temperature. Finally, the cells were washed and analyzed by flow cytometry.

### Fluorescence in situ hybridization

Fluorescence in situ hybridization (FISH) method was used for an analysis of chromosome alterations characteristic of CLL that are relevant markers of prognosis. The *locus*-specific probes for 11q22.3 (LSI *ATM*), 17p13.1 (LSI *TP53*), 13q.14.3 (D13S319), and the chromosome 12 centromere (CEP12) (Abbott Diagnostics) were used and FISH was performed according to the manufacturer’s instructions. Probes were denatured at 73 °C for 5 min and then applied to the determined areas on the slides. Following overnight hybridization at 37 °C, the slides were washed and air-dried in the darkness. Then, the slides were stained with DAPI and stored at −20 °C in the darkness. The samples were analyzed using the BX51 fluorescence microscope (OLYMPUS) and the CytoVision image analyzing system. At least 200 nuclei were analyzed for each probe with the cutoff value of 20 %.

### Clinical response to fludarabine-based treatment regimens

The assessment of clinical response to chemotherapy with fludarabine-based regimens was performed in the studied group of patients. We used the criteria of response to treatment proposed by WG-IWCLL in 2008 [13] based on WG-NCI criteria from 1996 [14]. According to these criteria, complete response requires the absence of symptoms and organomegaly, normal complete cell counts of peripheral blood, and less than 30 % of lymphocytes in bone marrow for at least 2 months. When size of the lymph nodes, spleen and liver, together with the peripheral blood data, were at least 50 % better than pre-treatment values, the partial response was achieved. Other patients were considered non-responders.

### Statistical analysis

The statistical analysis was performed using STATISTICA 10.0 software for Windows. We used Mann-Whitney *U* test for two-independent-group analyses. *p* < 0.05 was considered to be statistically significant.

## Results

### Active caspase-3 expression by CD19+/CD5+ peripheral blood and bone marrow cells in cultures with fludarabine

We detected active caspase-3 expression in malignant CD19+/CD5+ cells in all cell cultures from both peripheral blood and bone marrow after 24 h, similarly to our previously published results [12]. An increase in the percentage of caspase-3-positive cells was observed after 24 h in control cultures (cells without drugs) relative to the 0-h baseline control. This phenomenon was caused by spontaneous apoptosis of malignant cells in in vitro conditions, as it has been reported for CLL [15]. In cell cultures with fludarabine, the percentage of apoptotic cells after 24 h was obviously higher than at 0 h. It was also significantly higher than the percentage of apoptotic cells in control cultures without drugs after 24 h. The results (presented as the mean ± standard deviation) are shown in the Table 1 attached as a supplementary document.

### Assessment of chemotherapy response

The patients enrolled into the study were treated with the following fludarabine-based regimens:

- FC (fludarabine + cyclophosphamide), 11 persons
- TF (thalidomide + fludarabine), 5 persons
- FCR (fludarabine + cyclophosphamide + rituximab), 6 persons
- FCA (fludarabine + cyclophosphamide + alemtuzumab), 1 person

For all patients, these regimens were first-line treatment. Thirteen patients achieved complete remission, five partial remission, while five had no treatment response, what was an indication to change the treatment.

### Comparison of apoptosis rate in in vitro cultures with response to fludarabine-based chemotherapy

**Fig. 2:**
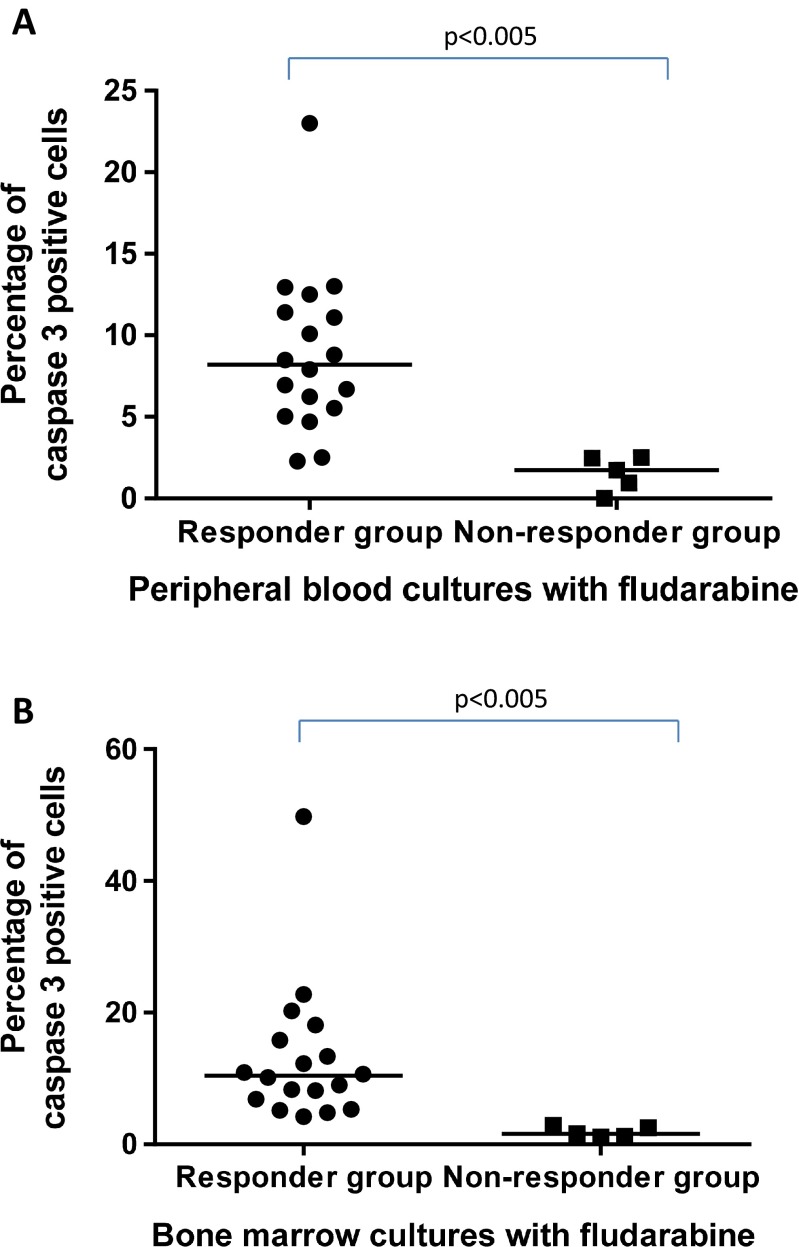
Percentage of caspase-3-positive leukemic CD19+/CD5+ cells in 24-h fludarabine-induced cell cultures in group of patients who clinically respond to fludarabine-based chemotherapy (*n* = 18) versus group of non-responders (*n* = 5). Data represent the drug-induced increase in the percentage of apoptotic cells above the respective values observed in control cultures of the same cell population. Statistically significant differences are indicated. **a** Peripheral blood samples. **b** Bone marrow samples

To assess the rate of apoptosis induced by fludarabine in the group of patients with clinical response to treatment and in the group of non-responders, we measured the expression of active caspase-3 in CD19+/CD5+ peripheral blood and bone marrow samples. Figure 2 illustrates the drug-induced increase in the frequency of apoptotic cells above the level of spontaneous apoptosis seen in the untreated 24-h parallel control cultures in both analyzed groups. The frequency of fludarabine-induced apoptosis in peripheral blood cultures of responding group was significantly higher than in non-responding one, and the same was true when compared with bone marrow cultures. Interestingly, among the patients who did not respond to chemotherapy, only in one case the presence of del17p and in one case del 11q were detected, while other persons had no genetic abnormalities. Similarly, only two persons were ZAP-70 positive and two were CD38+. The characteristic of non-responding patients is shown in Table 1.

**Table 1:** Clinical characteristic of patients who did not respond to the therapy based on fludarabine regimens

Patient number	Rai stadium	ZAP-70	CD38	Cytogenetic characteristic
1	4	−	−	No changes
2	4	+	+	No changes
3	2	+	+	del17p
4	2	−	−	No changes
5	1	−	−	del11q

## Discussion

Purine analogues are currently widely used in CLL patients. Three of them, fludarabine, pentostatin, and cladribine (2-CdA), were proved to have an effect on leukemic cells. They demonstrate antitumor activity in some ways such as direct interference with DNA and RNA synthesis, DNA repair mechanisms, induction of apoptosis, control of the cell cycle, and signal transduction pathways [16, 17]. Fludarabine remains the best studied compound of these three in CLL patients. Purine analogues have been reported to induce higher remission rates when they are employed as a first-line therapy compared with other treatment regimens containing alkylating agents or corticosteroids [18–21]. A major advance in CLL treatment was achieved by the combined use of different drugs. Thus, fludarabine-based therapies, in particular, its combination with other drugs such as cyclophosphamide or monoclonal antibody rituximab, were investigated in clinical trials [22, 23]. It has been proved that patients receiving fludarabine and rituximab had a better progression-free survival and overall survival than patients receiving fludarabine alone that represent a significant advance in CLL therapy [24, 25].

A number of markers of prognostic relevance have been identified for CLL patients, such as laboratory parameters reflecting the tumor burden or disease activity and markers related to the biology of the leukemia. The latter include genetic markers (like genomic aberrations, gene abnormalities (p53 and ATM), and the mutation status of the variable segment of immunoglobulin heavy chain genes (IgVH)) or surrogate markers for these factors such as ZAP-70 or CD38 [9, 25, 26]. Some of the prognostic factors can be used as predictors of the outcome of chemotherapy. Deletions of the short arm of chromosome 17 (del17p) are found in 5–8 % of chemotherapy-resistant patients. These deletions almost always include band 17p13, where the prominent tumor suppressor gene *TP53* is located. CLL patients carrying a del17p clone show marked resistance to genotoxic chemotherapies that cannot be overcome by the addition of anti-CD20 antibodies [7, 27]. Mutations of *TP53* are associated with very poor prognosis and are found in 4–37 % of patients with CLL [28]. The majority of patients with cytogenetically detected del17p show mutations in the remaining *TP53* allele. In cases without del17p, *TP53* mutations are much rarer, but have a similarly detrimental effect on chemotherapy response and overall survival [29]. However, the assessment of *TP53* mutation needs complicated and time-consuming methods, not widely available.

In the presented study, we tried to assess whether the rate of apoptosis caused by fludarabine in vitro can predict the outcome of chemotherapy in vivo. The experiments were performed in cultures of blood and bone marrow cells obtained from CLL patients prior to the onset of fludarabine-based treatment. The cultures were supplemented with fludarabine, and the percentage of caspase-3-positive cells after a 24-h period was determined. The results of the study showed statistically significant differences in apoptosis rate between the group of responders and non-responders as far as clinical response was concerned. The percentage of apoptotic cells was higher both in peripheral blood and bone marrow samples in the group of patients who obtained clinically complete or partial remission in comparison to the group with no response to fludarabine therapy. Interestingly, we observed that among the patients who did not respond to chemotherapy only in one case the presence of del17p and in one case del11q predicting purine analogues resistance, were detected. Other persons of this group had no genetic abnormalities. Nevertheless, it is true that we did not analyze the *TP53* mutations in the group of our patients. Such an analysis is elaborate, time-consuming, and impossible to perform in many hospitals. On the basis of our results, it can be concluded that quite easy to perform and simple assessment of apoptosis in drug-supplemented cultures may be also useful to predict treatment results. Furthermore, in our study, only two persons of the non-responders group expressed worse prognostic markers such as ZAP-70 and CD38, which indicates usefulness of the apoptosis test in predicting the outcome of therapy even in patients without expression of worse prognostic markers.

The seminal work in the field of in vitro prediction of clinical outcome in CLL was reported by Bosanquet et al. [30–32]. The authors used the differential staining cytotoxicity (DiSC) assay, as ex vivo apoptotic drug response test, to identify the sensitivity or resistance to fludarabine of lymphocytes from CLL patients. The results were compared with subsequent patient treatment, response, and survival. Treating fludarabine-test-resistant patients with fludarabine in vivo resulted in poor response and short survival compared with fludarabine-test-sensitive patients [30]. It was shown that fludarabine-test-resistance by DiSC assay is a powerful independent prognostic factor for CLL patients, which was in accordance with our results. The authors concluded that the DiSC assay could be considered a cost-effective guide to the treatment of chronic lymphocytic leukemia [31, 32].

Bromidge et al. [33] assessed the in vitro sensitivity of CLL cells to purine analogues and its correlation with clinical course in the group of 51 persons. Their results showed that the rate of apoptosis in cell cultures was comparable between group of patients who needed treatment and the group with stable disease. For this reason, such a test cannot be used to predict the time to start the therapy. Similarly to our study, the authors showed that sensitivity of leukemic cells to in vitro induced apoptosis correlated with clinical effects of therapy. Castejon et al. [34] analyzed fludarabine- and cladribine-induced apoptosis of CLL cells in vitro in comparison to in vivo response to chemotherapy with these drugs. The analysis was performed in the group of 50 CLL patients. Similarly to our results, in vitro apoptosis was in significant correlation with clinical response to treatment.

Żołnierczyk et al. [35] analyzed ex vivo sensitivity of leukemic cells obtained from CLL patients to conventional purine analogues and the selective CDK inhibitor R-roscovitine with and without the addition of an alkylating agent, prior to the onset of in vivo therapy. The kinetics and rate of spontaneous and drug-induced apoptosis of CLL cells under ex vivo conditions differed significantly between patients, reflecting the variability observed during in vivo treatment. Similarly to our results, the authors concluded that ex vivo testing might be useful for identifying the most potent first-line therapeutic regimen for specific CLL patients and possibly for the design of therapies tailored for individual CLL patients [35]. We showed additionally that sensitivity of leukemic cells to purine analogues in vitro seemed to be independent of cytogenetic changes detected in cells.

Recently, it has been reported that not only drug-induced apoptosis but also spontaneous programmed death may be a simple prognostic test which can predict the course of the disease and response to treatment. Witkowska et al. [36] compared the level of spontaneous apoptosis with prognostic factors and clinical course of the disease in 135 treatment naive patients with CLL. Rate of apoptosis in patients with stable disease was found to be significantly higher than in the group with progressive course of the disease. Furthermore, the level of apoptosis correlated inversely with the progression-free survival.

In conclusion, the obtained results indicate that the sensitivity of CLL cells to fludarabine induced apoptosis in in vitro conditions may reflect the effects of these drugs in vivo. Assessment of drug-induced apoptosis in cell cultures seems to be an easy to perform and simple test predicting the outcome of chemotherapy with fludarabine CLL patients.

## Electronic supplementary material

Table 1 (DOC 33 kb)
