# Supplementary material for: The rate of in vitro fludarabine-induced peripheral blood and bone marrow cell apoptosis may predict the chemotherapy outcome in patients with chronic lymphocytic leukemia
Source: Eur J Clin Pharmacol. 2015 Jul 5;71(9):1121–7. doi: 10.1007/s00228-015-1893-0 (PMC4532719; doi:10.1007/s00228-015-1893-0)
Supplement: Supplementary file 1 — (DOC 33 kb) [file 228_2015_1893_MOESM1_ESM.doc]

Table 1:

The active caspase 3 expression by CD19+/CD5+ cells in *in vitro* cultures of peripheral blood and bone marrow obtained from untreated CLL patients with fludarabine added. Values represent the mean ± standard deviation.

|  |  | **Blood** | **Bone marrow** | **Percentage of caspase 3 positive CD19+/CD5+ cells** |
| --- | --- | --- | --- | --- |
| **Control** |  | 15.27 ±10.52 | 19.19±10,20 |  |
| **Fludarabine** |  | 23.30±15.30 | 30.54±17.42 |  |
| **Fludarabine**  **(above the control level)** | **Whole group** | 8.25±9.18 | 11,10±10.51 |  |
|  | **Responder group** | 10.12±9.58 | 13.11±10.52 |  |
|  | **Non Responder Group** | 1.53±1.06 | 2.03±0.86 |  |
